# Data on body weight and liver functionality in aged rats fed an enriched strawberry diet

**DOI:** 10.1016/j.dib.2017.06.021

**Published:** 2017-06-16

**Authors:** Francesca Giampieri, Josè M. Alvarez-Suarez, Massimiliano Gasparrini, Tamara Y. Forbes-Hernandez, Sadia Afrin, Corrado Rubini, Antonio Zizzi, Josè L. Quiles, Bruno Mezzetti, Maurizio Battino

**Affiliations:** a Dipartimento di Scienze Cliniche Specialistiche ed Odontostomatologiche (DISCO)-Sez. Biochimica, Facoltà di Medicina, Università Politecnica delle Marche, Via Ranieri 65, 60131 Ancona, Italy; b Escuela de Medicina Veterinaria y Zootecnia, Facultad de Ciencias de la Salud, Universidad de Las Américas (UDLA), Jose Queri, Quito 170125, Ecuador; c Area de Nutrición y Salud, Universidad Internacional Iberoamericana (UNINI), Calle 15, 24560 Campeche, Mexico; d Dipartimento di Scienze Biomediche e Sanita׳ Pubblica, Sez. Anatomia Patologica, Università Politecninca delle Marche, Via Conca 71, 60126 Ancona, Italy; e Departamento de Fisiologia, Instituto de Nutrición y Tecnología de los Alimentos ‘‘José Mataix”, Centro de Investigaciones Biomedicas, Universidad de Granada, 18100 Granada, Spain; f Dipartimento di Scienze Agrarie, Alimentari e Ambientali, Università Politecnica delle Marche, Via Ranieri 65, 60131 Ancona, Italy; g Centre for Nutrition & Health, Universidad Europea del Atlantico (UEA), C/Isabel Torres 21, 39011 Santander, Spain

**Keywords:** Strawberry consumption, Aging, Body weight, Liver functionality

## Abstract

Here, we present new original data on the effects of strawberry consumption on body weight and liver status of aged rats. Wistar rats aged 19–21 months were fed a strawberry enriched diet prepared by substituting 15% of the total calories with freeze-dried strawberry powder for two months. Body weight, plasma biomarkers of liver injury (alanine transferase, aspartate aminotransferase and alkaline phosphatase) and liver histological analysis were assessed. These data indicate that strawberry supplementation did not interfere with normal animal maintenance and with liver structure and functionality. For further details and experimental findings please refer to the article “Strawberry consumption improves aging-associated impairments, mitochondrial biogenesis and functionality through the AMP-Activated Protein Kinase signaling cascade” in FOOD CHEMISTRY (Giampieri et al., 2017) [1].

**Specifications Table**

**Table:** 

Subject area	*Medicine*
More specific subject area	*Nutritional biochemistry, aging*
Type of data	*Tables, images, file text*
How data was acquired	*Absorbance was acquired using a microplate reader (Bio-Tek Instrument Co.,WA, USA), while tissue observation was performed with APERIO ScanScope digital system (Nikon, Firenze, Italy).*
Data format	*Raw data collection and analysis*
Experimental factors	*Plasma isolation and tissue staining*
Experimental features	*Body weight, plasma biomarkers of liver injury and liver histological analysis were performed in aged rats after two months of strawberry supplementation.*
Data source location	*Ancona, Italy*
Data accessibility	*Data are available with this article*
Related research article	*Giampieri F et al. Strawberry consumption improves aging-associated impairments, mitochondrial biogenesis and functionality through the AMP-Activated Protein Kinase signaling cascade. Food Chemistry, In press.*

**Value of the data**

• The presented data indicate that strawberry consumption doesn’t increase body weight and liver ratio.
• The presented data show that strawberry consumption doesn’t affect the structure and functionality of aged livers.
• These data are the further evidence that strawberries can be used as a natural source of bioactive compounds with healthy benefits.
• These data could be of utmost importance to promote these fruits also in the diet of aged people.

## 1 Data

**Fig. 1:**
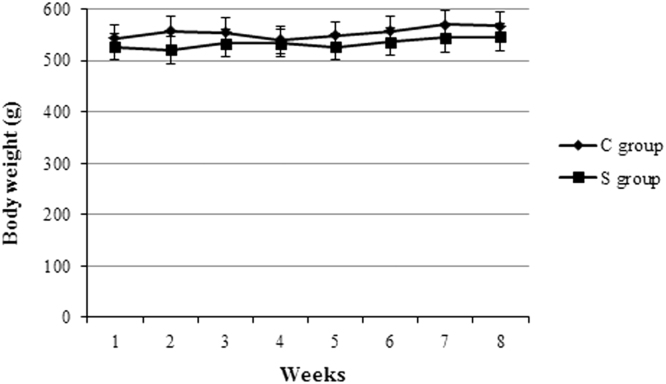
Strawberry consumption did not interfere with animal weight. Rats were fed a standard diet (C group) or an enriched strawberry diet (S group) for 2 months. The animals were weighed once a week for the whole experimental period.

**Fig. 2:**
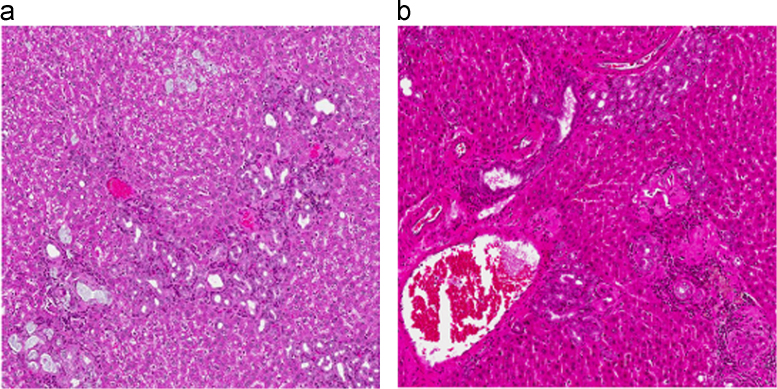
Representative liver histological analysis of rats fed with standard diet (a) and strawberry diet (b).

Rats were fed an enriched strawberry diet for two months and were weighed once a week for the whole experimental period. Compared with control group, strawberry supplementation didn’t interfere with body weight (Fig. 1) and liver ratio (Table 1). In addition, strawberry consumption didn’t affect biomarkers of liver injury measured in plasma (Table 1) as well as liver histology (Fig. 2): no differences were in fact observed for these parameters in the group supplemented with strawberries compared to the control group.

**Table 1:** Strawberry consumption did not affect liver functionality. Rats were fed a standard diet (C group) or an enriched strawberry diet (S group) for 2 months. The liver ratio (%) was calculated as g/100 g body weight; ns: not significant. (ALT, alanine transferase; AST, aspartate aminotransferase; ALP, alkaline phosphatase).

	Liver ratio (% body weight)	ALT IU/L	AST IU/L	ALP IU/L
C group	2.87±0.01^ns^	9.11±0.33^ns^	59.96±1.36^ns^	89.73±2.32^ns^
S group	2.89±0.02^ns^	8.79±0.16^ns^	61.5±2.01^ns^	87.79±1.21^ns^

## 2 Experimental design

Wistar rats (Rattus norvegicus, 19–21 months, 500–550 g) provided by the “Istituto Nazionale di Ricovero e Cura per gli Anziani” (INRCA, Ancona, Italy), were housed individually and maintained on a 12 h light/12 h darkness cycle with free access to drinking water. The animals randomly received either a standard diet (C group, *n*=8) or a strawberry-enriched diet (S group, *n*=8) (Table 2) for 2 months. Both diets were supplied in the form of powder and daily prepared by mixing each individual ingredient using a rotating mixer and kept in the dark at a temperature of 4 °C. Compared to the standard diet (AIN93M), the strawberry enriched diet was prepared by substituting 15% of the total calories with freeze-dried strawberry powder (Table 2, Table 3), and the amount of macro- and micronutrient adjusted to be identical between the two diets. The animals received their respective food and drink at libitum. Rats were weighed once a week for the whole experimental period.

**Table 2:** Composition of standard (AIN-93M) and strawberry-enriched diet (g/1000 g).

Ingredients	Standard diet	Strawberry diet
Cornstarch	465.70	465.70
Casein	140.00	132.00
Dextrinized cornstarch	155.00	155.00
Sucrose	100.00	0
Soybean oil	40.00	31.00
Fiber	50.00	17.00
Mineral Mix	35.00	35.00
Vitamin Mix	10.00	10.00
L-Cystine	1.80	1.80
Choline bitartrate	2.50	2.5
Strawberry	0	150
Total	1000	1000

**Table 3:** Nutrient composition, phytochemical content and antioxidant capacity of strawberry powder.

**Parameter**		**Quantification**
Vitamin C (mg/g)		4.70±0.40
Total phenolic (mg/g)		21.30±0.00
Total flavonoid (mg/g)		8.00±0.01
Anthocyanins (mg/g)		
	Cy-3-glucoside	0.31±0.00
	Cy 3-malonylglucoside	0.09±0.00
	Pg 3-glucoside	3.97±0.03
	Pg 3-rutinoside	0.39±0.00
	Pg 3-malonylglucoside	0.67±0.00
	Pg 3-acetylglucoside	0.04±0.00
	Pg 3-succinylarabinose	0.05±0.00
TAC (mM TE)		
	FRAP	0.42±0.00
	TEAC	1.21±0.03

## 3 Materials and methods

At the end of the two months of strawberry supplementation, the rats were anesthetized with 4% isoflurane inhalation and blood was collected by intra-cardiac puncture and immediately transferred into heparin-containing tubes. Heparinized plasma was isolated by centrifugation at 1130 g for 20 min at 15 °C and stored at −80 °C until analyses. After exsanguination, the whole livers were carefully removed, washed with ice-cold 0.9% NaCl solution, weighed and preserved in formaldehyde for histological analysis [1].

### 3.1 Plasma analysis

Plasma levels of biomarkers of liver injury (alanine transferase, aspartato aminotransferase and alkaline phosphatase) were determined by commercial kits (Spinreact, St. Esteve d׳en Bas, Girona, Spain) according to manufacturer׳s instructions, using a microplate reader (Bio-Tek Instrument Co.,WA, USA) as previously reported [2].

### 3.2 Liver histological analysis

Rat livers were dissected immediately and preserved in 10% buffered formaldehyde at room temperature for microscopic observations. Tissue samples were embedded in paraffin and 4–6 μm sections were cut using a rotary microtome and stained with hematoxylin and eosin. Histological evaluation was made in liver tissues by an expert pathologist with a microscope at 40×. Hematoxylin and eosin stained slides were scanned with APERIO ScanScope digital system (Nikon, Firenze, Italy) and representative images were recorded with ImageScope software (Nikon) at the original magnification of 10×.

## References

[1] Giampieri F., Alvarez-Suarez J.M., Cordero M.D., Gasparrini M., Forbes-Hernandez T.Y., Afrin S., Santos-Buelga C., González-Paramás A.M., Astolfi P., Rubini C., Zizzi A., Tulipani S., Quiles J.L., Mezzetti B., Battino M. (2017). Strawberry consumption improves aging-associated impairments, mitochondrial biogenesis and functionality through the AMP-Activated Protein Kinase signaling cascade. Food Chem..

[2] Giampieri F., Alvarez-Suarez J.M., Gasparrini M., Forbes-Hernandez T.Y., Afrin S., Bompadre S. (2016). Strawberry consumption alleviates doxorubicin-induced toxicity by suppressing oxidative stress. Food Chem. Toxicol..

